# T2‐weighted imaging of rectal cancer using a 3D fast spin echo sequence with and without deep learning reconstruction: A reader study

**DOI:** 10.1002/acm2.70031

**Published:** 2025-02-20

**Authors:** Dan Nguyen, Sarah Palmquist, Ken‐Pin Hwang, Jingfei Ma, Usama Salem, Jia Sun, Xinzeng Wang, Jong Bum Son, Randy Ernst, Peng Wei, Harmeet Kaur, Nir Stanietzky

**Affiliations:** ^1^ Department of Radiology The University of Texas MD Anderson Cancer Center Houston Texas USA; ^2^ Department of Biostatistics The University of Texas MD Anderson Cancer Center Houston Texas USA; ^3^ GE HealthCare Global MR Applications and Workflow Houston Texas USA

**Keywords:** 3‐dimensional magnetic resonance imaging, deep learning reconstruction, rectal cancer

## Abstract

**Purpose:**

To compare image quality and clinical utility of a T2‐weighted (T2W) 3‐dimensional (3D) fast spin echo (FSE) sequence using deep learning reconstruction (DLR) versus conventional reconstruction for rectal magnetic resonance imaging (MRI).

**Methods:**

The study included 50 patients with rectal cancer who underwent rectal MRI consecutively between July 7, 2020 and January 20, 2021 using a T2W 3D FSE sequence with DLR and conventional reconstruction. Three radiologists reviewed the two sets of images, scoring overall SNR, motion artifacts, and overall image quality on a 3‐point scale and indicating clinical preference for DLR or conventional reconstruction based on those three criteria as well as image characterization of bowel wall layer definition, tumor invasion of muscularis propria, residual disease, fibrosis, nodal margin, and extramural venous invasion.

**Results:**

Image quality was rated as moderate or good for both DLR and conventional reconstruction for most cases. DLR was preferred over conventional reconstruction in all of the categories except for bowel wall layer definition.

**Conclusion:**

Both conventional reconstruction and DLR provide acceptable image quality for T2W 3D FSE imaging of rectal cancer. DLR was clinically preferred over conventional reconstruction in almost all categories.

## 1 INTRODUCTION

Rectal cancer is among the most common malignant neoplasms worldwide and accounts for a significant portion of cancer‐related morbidity and mortality.^1^ The treatment strategy for patients with rectal adenocarcinoma—specifically, the need for chemotherapy, radiotherapy, and/or surgery—is highly dependent on and often guided by locoregional staging.^2^ Rectal magnetic resonance imaging (MRI) is an essential tool to accurately characterize the local extent or stage of the tumor and enable clinicians to plan initial and subsequent therapy.^1^, ^2^ Additionally, rectal MRI provides useful information for predicting rectal cancer survival outcomes.^3^ In interpreting rectal MRI, it is important to define the anatomic relationship between the tumor, mesorectal fascia, sphincter complex, and adjacent organs. Consequently, obtaining diagnostic images of adequate quality is critical for a successful rectal MRI study.^3^

Multiplanar high‐resolution T2‐weighted (T2W) imaging is an essential component of rectal cancer MRI, both in the initial diagnosis/staging and the assessment of treatment response. Currently, the 2‐dimensional (2D) fast spin echo (FSE) or turbo spin echo (TSE) pulse sequence (herein referred to FSE) is the most commonly used sequence for T2W imaging of the rectum.^4^ Although 2D FSE is a more established technique in rectal MRI, it must be repeated for each of the planes (axial, coronal, and sagittal).^4^ Depending on tumor anatomy and orientation, additional acquisitions in an oblique plane may also be needed, which frequently requires an in‐study review by a radiologist and communication with the imaging technologists, presenting a hurdle in the workflow and prolonging the study table time.

In contrast, 3‐dimensional (3D) sequences acquire data volumetrically by applying an additional set of magnetic field gradients along the slice direction, which encodes the acquired signals and allows spatial localization in the slice direction similarly to that of phase encoding in 2D sequences. A 3D Fourier transform is applied to the signals to transform the acquired data in the 3D k‐space into images of the different contiguous slices simultaneously.^5^, ^6^, ^7^

3D FSE is therefore able to achieve a higher effective spatial resolution in the slice direction than 2D FSE. While 3D encoding can increase scan time considerably, the data acquisition scheme of 3D FSE is synergistic with parallel imaging acceleration and can achieve scan times comparable to those with 2D FSE. In summary, the beneficial salient features of 3D FSE which differentiate it from 2D FSE include slice contiguity, higher effective slice resolution, and higher SNR efficiency.^4^, ^7^

Clinically, 3D FSE is now routinely used in high‐resolution imaging of relatively static anatomy, such as the brain and knee, but has not been commonly used in rectal MRI.^7^ In recent years, deep learning technology has become more widely used due to advances in network design and semiconductor technology. Most of today's deep learning reconstruction (DLR) is based on supervised learning models that are implemented using a convolutional neural network (CNN).^8^ The CNN is trained using noisy input data, which are compared with high‐SNR ground truth images. After enough training, the CNN can reconstruct noisy data into new images that are similar to the ground truth data set. Image denoising is one of the first and most impactful applications of DLR in MRI and has demonstrated the ability to improve SNR at the reconstruction stage.^9^ Thus, DLR offers improved SNR without the cost of modifying other parameters such as scan time or field strength, making DLR a fundamentally important tool for current and future clinical use. Recently, DLR was shown to improve image quality in many 2D applications.^10^, ^11^, ^12^, ^13^ However, clinical implementation of 3D DLR has been limited.^14^ In this work, we conducted a reader study and investigated the use of 3D DLR^15^ for rectal MRI in patients with a pathologically proven diagnosis of rectal adenocarcinoma and no prior rectal surgery and assessed its impact on diagnostic quality and the potential clinical adoption of the combined technique.

## 2 METHOD

We retrospectively identified 73 patients who had undergone rectal MRI consecutively from 7 July, 2020 to 20 January, 2021 in our imaging database. Patients with nonadenocarcinoma rectal tumors, such as anorectal melanoma, squamous cell carcinoma, and carcinoid tumor, were excluded. Those with prior rectal surgery such as total colectomy, abdominal perineal resection, and low anterior resection were also excluded. Additionally, patients with metallic implants such as rectal stents were excluded due to artifacts, and studies performed for proctitis, fistula, or recurrence outside the rectum were not included. In total, 23 patients were excluded based on the above criteria. The remaining 50 patients were all referred for a clinical MRI exam with an indication of initial staging, treatment response, or surveillance for rectal adenocarcinoma.

All the MRI exams were performed on either a 3T or a 1.5T MR scanner (GE Healthcare, Waukesha, WI) running on software version DV26.0, using an external torso array coil. Thirty nine patients were scanned on 3T and 11 patients were scanned on 1.5T. The 3D FSE imaging was acquired with a vendor‐proprietary Cube sequence.^6^ The typical scan parameters for both 1.5T and 3T were as follows: TR/TE = 2500/132 ms, echo train length = 100, field of view = 18 cm, acquisition matrix = 320 × 256, acquired slice thickness = 3.0 mm, reconstructed slice thickness = 1.5 mm, acceleration factor = 2.0 (phase) × 1.0 (slice), number of slices = 72, acquisition time = 3 min and 56 s.^6^ In addition to the images obtained by the conventional reconstruction, we applied a proprietary DLR algorithm trained to reduce noise and Gibbs ringing to reconstruct an additional set of images from the same raw data for comparison.^16^

Three board‐certified radiologists (NS, US, SP with 23, 16, 8 years of experience in abdominal imaging) reviewed and evaluated the conventional and DLR images in a nonblinded fashion. Using a 3‐point scale (1: good, 2: moderate, 3: poor) and based on their clinical experience in reading rectal cancer MRIs, each radiologist independently scored the images on overall SNR, motion artifacts, and overall image quality, as well as the characterization of the following measures of clinical utility: definition of bowel wall layers, tumor invasion of muscularis propria, residual disease, fibrosis, nodal margins, and extramural venous invasion. Further, each radiologist compared the conventional and DLR images and recorded their preferred image for each of the above categories. A tie was recorded if there was no preference. A small number (a maximum of 6%) of exams were not scored in individual particular categories as they were not applicable due to personal assessment or diagnostic uncertainty, which varied between individual readers. This small number did not influence the statistical analysis.

Summary statistics of the image quality evaluations were performed using frequency tables and percentages. Fleiss's kappa statistics was used to assess agreement between the three readers. The proportion of DLR images preferred was estimated with a 95% confidence interval by the method of Clopper and Pearson.^17^ We tested the null hypothesis that the proportion of DLR preferred or equally preferred is less than 50% using the one‐sided exact binomial test. Statistical analysis was carried out using R (version 3.6.3, R Development Core Team).

## 3 RESULTS

The demographics of the study patients is presented in Table 1.

**TABLE 1:** Patient age, gender and tumor stage.

Age in years, mean (range)	56 (29–81)
**Gender, N (%)**	
Male	26 (53%)
Female	23 (47%)
**Tumor staging, N (%)**	
T1	5 (10%)
T2	4 (8%)
T3	21 (43%)
T4	19 (39%)

On an absolute scale, all three readers rated both the DLR and conventional images as moderate or good in overall quality for most cases. On a relative scale, all three readers rated DLR images as preferred over or equal to (tie) conventional images in > 85% of the cases in the fibrosis, motion, nodal margin, and SNR categories (Table 2). In particular, DLR performed exceptionally well in the nodal margin category. Fair to moderate inter‐reader agreement was found in the evaluation of all categories except for motion artifacts (kappa = 0.15), tumor invasion of muscularis propria (kappa = 0.11), and fibrosis (kappa = −0.07) (Table 3).

**TABLE 2:** Reader preference for assessing the morphologic tumor characteristics.

	Reader 1			Reader 2			Reader 3		
	#DLR preferred or tie/Total	Proportion^a^ (95% CI)	P‐value^b^	#DLR preferred or tie/Total	Proportion^a^ (95% CI)	P‐value^b^	#DLR preferred or tie/Total	Proportion^a^ (95% CI)	P‐value^b^
**Clear definition of bowel wall layers**	32/47	0.68 (0.53,0.81)	0.009	24/48	0.5 (0.35,0.65)	0.56	24/47	0.51 (0.36,0.66)	0.5
**Extramural venous invasion**	13/13	1 (0.75,1)	<0.001	23/27	0.85 (0.66,0.96)	<0.001	12/14	0.86 (0.57,0.98)	0.006
**Fibrosis**	20/21	0.95 (0.76,1)	<0.001	31/35	0.89 (0.73,0.97)	<0.001	17/19	0.89 (0.67,0.99)	<0.001
**Motion**	48/50	0.96 (0.86,1)	<0.001	48/49	0.98 (0.89,1)	<0.001	44/48	0.92 (0.8,0.98)	<0.001
**Nodal margin**	20/22	0.91 (0.71,0.99)	<0.001	32/33	0.97 (0.84,1)	<0.001	17/18	0.94 (0.73,1)	<0.001
**Overall image quality**	46/50	0.92 (0.81,0.98)	<0.001	43/48	0.9 (0.77,0.97)	<0.001	43/48	0.9 (0.77,0.97)	<0.001
**Overall SNR**	45/50	0.9 (0.78,0.97)	<0.001	46/49	0.94 (0.83,0.99)	<0.001	44/48	0.92 (0.8,0.98)	<0.001
**Residual disease**	20/24	0.83 (0.63,0.95)	<0.001	34/36	0.94 (0.81,0.99)	<0.001	22/24	0.92 (0.73,0.99)	<0.001
**Tumor invasion of muscularis propria**	33/39	0.85 (0.69,0.94)	<0.001	35/41	0.85 (0.71,0.94)	<0.001	30/40	0.75 (0.59,0.87)	0.001

^a^ The proportion is defined as the number of DLR preferred or tied divided by the total.

^b^ The *p*‐value is derived from a one‐sided exact binomial test of the null hypothesis that the proportion of DLR preferred or equally preferred is less than 50%.

**TABLE 3:** Statistical analysis of reader agreement on morphologic tumor characteristics.

Reader agreement by Fleiss's kappa.		
	Fleiss's kappa	*p*‐value
**Clear definition of bowel wall layers**	0.39	<0.001
**Extramural venous invasion**	0.21	0.185
**Fibrosis**	0.44	<0.001
**Motion**	0.10	0.235
**Nodal margin**	0.29	0.031
**Overall image quality**	0.21	0.012
**Overall SNR**	0.55	<0.001
**Residual disease**	0.36	0.003
**Tumor invasion of muscularis propria**	0.39	<0.001

**FIGURE 1:**
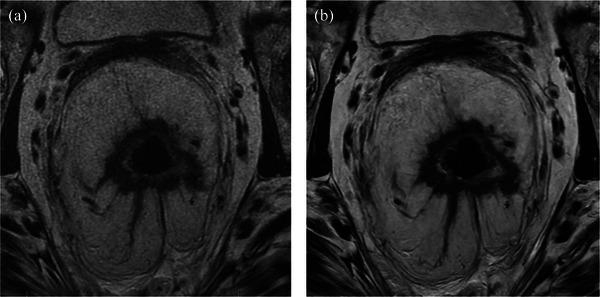
Pre‐treatment magnetic resonance imaging of a 67‐year‐old woman with cT3N2M0 rectal adenocarcinoma using 3‐dimensional fast spin echo with (A) conventional reconstruction and (B) deep learning reconstruction (DLR). The images demonstrate a circumferential mid‐rectal tumor with extensive extramural venous invasion and extension beyond the muscularis propria. DLR was preferred by all three radiologists for overall image quality and signal‐to‐noise ratio.

**FIGURE 2:**
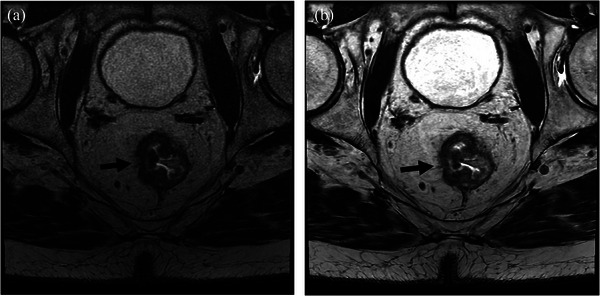
Pre‐treatment pelvic magnetic resonance imaging of an 82‐year‐old man with rectal adenocarcinoma using 3‐dimensional fast spin echo with (A) conventional reconstruction and (B) deep learning reconstruction (DLR). The images demonstrate a near‐circumferential mid‐rectal tumor which extends beyond the muscularis propria into the perirectal fat (black arrow). The readers preferred DLR over conventional reconstruction in this clinical utility category due to the improved image quality.

DLR was preferred over the conventional reconstruction image for overall image quality and SNR, for example as illustrated in the case shown in Figure 1. Additionally, DLR helped to better characterize tumor invasion through the muscularis propria (Figure 2). DLR was preferred in more than 50% of cases by all three reviewers in all categories except definition of the bowel wall layers (Table 2).

## 4 DISCUSSION

In this work, we evaluated the quality and potential clinical value of T2W imaging of rectal cancer with a 3D FSE sequence after applying a DLR algorithm and compared those images to images that were reconstructed using the conventional algorithm. Our readers agreed that the DLR algorithm generally resulted in better overall image quality and SNR. Additionally, there were multiple clinical utility categories where DLR was preferred, such as characterizing tumor invasion through the muscularis propria, an important upstaging feature. Overall, these findings suggest that 3D T2W images (either DLR or conventional) are acceptable for rectal cancer imaging.

**FIGURE 3:**
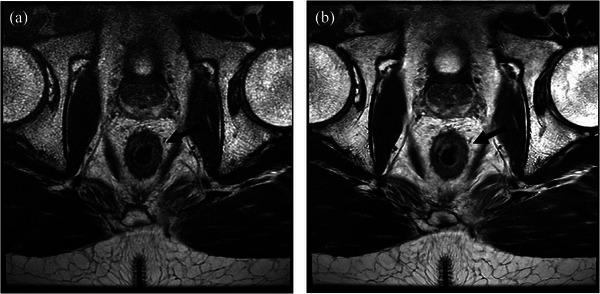
Post‐treatment pelvic magnetic resonance imaging of a 45‐year‐old man with rectal adenocarcinoma using 3‐dimensional fast spin echo with (A) conventional reconstruction and (B) deep learning reconstruction (DLR). The DLR images show a smoother interface between the bowel wall layers (mucosa/submucosa/muscularis propria), but the conventional images are sharper and better delineate these layers (arrow). The readers did not prefer DLR in this clinical utility category.

Although DLR was generally preferred, it was not preferred for imaging of bowel wall definition. For example, in some cases, it was difficult to differentiate the bowel wall layers on the 3D FSE images with DLR when compared to the 3D FSE images using conventional reconstruction (Figure 3). We theorize that this may be attributable to the denoising effect of DLR in the presence of bowel motion, which can result in the blurring of sharp edges and thus make it more difficult to differentiate anatomy such as bowel wall layers. DLR can be applied at less aggressive settings which would result in images more similar to the conventional reconstruction images, but these lower settings were not evaluated in this study. Although the boundaries of the bowel wall layers were more difficult to delineate with DLR, the denoising was found to be helpful in estimating whether tumor extended beyond the muscularis propria.

3D acquisition allows for reconstruction at slices that are generally thinner and with more uniform slice profile than a 2D acquisition. This aids multiplanar reformatting during post‐processing, resulting in mitigation of the “stair‐step” artifact commonly seen when reformatting stacks of 2D images. Since the true slice thickness of our 3D FSE protocol was 3 mm, the reformatted planes in our study would not have the same resolution as in the native plane. However, 3D FSE images can be acquired with thinner slices to potentially achieve diagnostic quality in the other planes. While this comes at the expense of increased acquisition time, the sequence can be acquired without requiring in‐study feedback from an on‐call radiologist, and would also be faster than acquiring images in all three imaging planes separately. Thus, 3D FSE offers more flexibility as well as a possible reduction in both active scan time and overall table time, potentially improving scanner and patient throughputs.^7^

There were several limitations in our study. First, 3D FSE with DLR was compared against the same 3D FSE data without DLR, but was not compared directly with 2D FSE, which is the current standard for rectal cancer imaging at most institutions.^4^ Thus, this study demonstrates the potential clinical utility of 3D FSE DLR but not its superiority in diagnostic performance over the current standard of care. Second, the imaging parameters of the 3D FSE sequence were designed for diagnostic imaging without the use of DLR. The performance of DLR on an accelerated or higher‐resolution 3D FSE sequence was not evaluated. Third, all MRIs were acquired on a mixed 3T and 1.5T scanners from a single vendor. It is possible that there may have been some differences in image quality for scanners of different field strength, and DLR algorithms may vary between manufacturers. Fourth, the assessments were not compared to the pathologic specimens and therefore an objective analysis of whether the smoothing techniques employed by DLR lead to inaccurate interpretations cannot be assessed. Fifth, both 1.5T and 3T MR scanners were utilized and whether the performance of the DLR data differed significantly between the two field strengths was not studied. We considered that the number of patients in the study was insufficient to perform a statistical analysis to determine any potential differences in image quality and clinical utility that may or may not exist between the two field strengths. Sixth, our study is limited as a subjective reader study to determine the clinical impact of 3D imaging with DLR. We did not perform a quantitative image analysis as it is beyond the scope of our study purpose. Further, determining some traditionally used image metrics such as SNR and CNR is poorly defined or can be challenging in the context of DLR and other imaging setup and options used in the acquisition. Finally, the readers were not blinded as to whether DLR was employed when reviewing the studies, potentially introducing a rating bias.

Future studies are needed to compare the accuracy of 3D DLR to conventional two‐dimensional imaging to ensure that it is at least equivalent. In addition, studies that compare 3D DLR to the gold standard pathologic specimens are necessary to verify that the DLR algorithm, while boosting SNR and aiding interpretation, does not falsely modify the tumor stage. Studies could also be performed to compare 1.5T and 3T MR scanners with and without DLR to assess whether the performance is field strength dependent. Finally, a quantitative rather than qualitative image analysis study could be undertaken to supplement this subjective reader study.

## 5 CONCLUSION

Our study demonstrated that DLR provides improved overall image quality and clinical utility of 3D FSE over conventional reconstruction in the T2W imaging of rectal cancer. While the current workhorse of T2W imaging of rectal cancer is 2D FSE due to its superior SNR and contrast resolution, 3D FSE offers advantages in multiplanar reconstruction and flexibility in protocol design. Thus, any significant improvement to 3D FSE introduced by the DLR algorithm provides an opportunity to reconsider the current standard of imaging. Future studies will need to compare 3D FSE with the current standard of 2D FSE in the T2W imaging of rectal cancer.

## AUTHOR CONTRIBUTIONS

Dan Nguyen, Nir Stanietzky, Sarah Palmquist, Ken‐Pin Hwang, Jingfei Ma, and Jong Bum Son contributed to the data collection, data analysis, writing, and editing the manuscript. Usama Salem and Xinzeng Wang contributed to the data collection and analysis. Jong Bum Son and Peng Wei contributed to the statistical analysis of the data. Randy Ernst and Harmeet Kaur contributed to the manuscript editing.

## CONFLICT OF INTEREST STATEMENT

J.M.: Consultant for C4 Imaging, LLC; and IP licensing to GE Healthcare and to Siemens Healthineers. The authors thank Dawn Chalaire, senior scientific editor, Research Medical Library, for editing the manuscript. This study was supported in part by the NIH/NCI under award number P30CA016672.
